# Antibacterial Synthetic Nanocelluloses Synergizing with a Metal-Chelating Agent

**DOI:** 10.1021/acsabm.3c00846

**Published:** 2023-11-15

**Authors:** Takeshi Serizawa, Saeko Yamaguchi, Kai Sugiura, Ramona Marten, Akihisa Yamamoto, Yuuki Hata, Toshiki Sawada, Hiroshi Tanaka, Motomu Tanaka

**Affiliations:** † Department of Chemical Science and Engineering, School of Materials and Chemical Technology, Tokyo Institute of Technology, 2-12-1 Ookayama, Meguro-ku, Tokyo 152-8550, Japan; ‡ Physical Chemistry of Biosystems, Institute of Physical Chemistry, Heidelberg University, Heidelberg D69120, Germany; § Center for Integrative Medicine and Physics, Institute for Advanced Study, Kyoto University, Kyoto 606-8501, Japan

**Keywords:** cellulose oligomer, crystalline assembly, antibacterial cationic polymer, bactericidal activity, ethylenediaminetetraacetic acid, synergistic effect

## Abstract

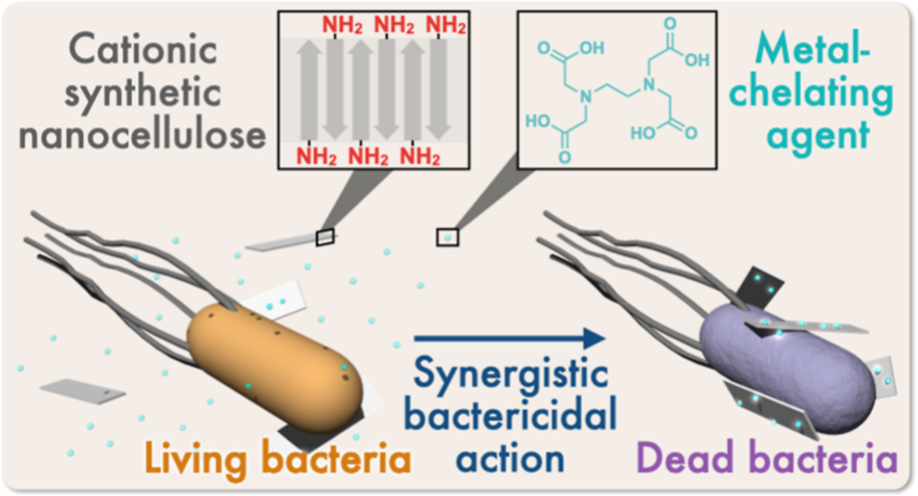

Antibacterial materials composed of biodegradable and biocompatible constituents that are produced via eco-friendly synthetic strategies will become an attractive alternative to antibiotics to combat antibiotic-resistant bacteria. In this study, we demonstrated the antibacterial properties of nanosheet-shaped crystalline assemblies of enzymatically synthesized aminated cellulose oligomers (namely, surface-aminated synthetic nanocelluloses) and their synergy with a metal-chelating antibacterial agent, ethylenediaminetetraacetic acid (EDTA). Growth curves and colony counting assays revealed that the surface-aminated cellulose assemblies had an antibacterial effect against Gram-negative *Escherichia coli* (*E. coli*). The cationic assemblies appeared to destabilize the cell wall of *E. coli* through electrostatic interactions with anionic lipopolysaccharide (LPS) molecules on the outer membrane. The antibacterial properties were significantly enhanced by the concurrent use of EDTA, which potentially removed metal ions from LPS molecules, resulting in synergistic bactericidal effects. No antibacterial activity of the surface-aminated cellulose assemblies was observed against Gram-positive *Staphylococcus aureus* even in the presence of EDTA, further supporting the contribution of electrostatic interactions between the cationic assemblies and anionic LPS to the activity against Gram-negative bacteria. Analysis using quartz crystal microbalance with dissipation monitoring revealed the attractive interaction of the surface-aminated cellulose assembly with LPS Ra monolayers artificially produced on the device substrate.

## 1 Introduction

Antibacterial materials that suppress the uncontrolled growth of pathogenic bacteria have attracted considerable attention for preventing bacterial infections or mitigating bacterial virulence in the fields of food, cosmetics, and medicine.^1−4^ Due to the unfortunate evolution of antibiotic-resistant bacteria,^5−8^ antibacterial synthetic polymers have been developed as alternatives to antibiotics.^9−11^ Advantages of antibacterial synthetic polymers include designability of the chemical structure, stability under biological conditions, processability, and low skin penetration compared to low-molecular-weight organic or inorganic antibacterial materials. Antibacterial synthetic polymers typically have cationic and hydrophobic functionalities (e.g., amino and alkyl groups, respectively), which have been designed with inspiration from cationic host defense peptides^12^ or biocidal cationic surfactants (e.g., benzalkonium chlorides).^13^ Cationic and hydrophobic groups of polymers interact electrostatically and hydrophobically with the anionic lipid bilayer of bacteria, disrupting the membrane structure and thus killing bacteria. An emerging alternative polymer design is the combination of cationic and hydrophilic functionalities.^14−16^ Because of the limited attractive interactions between cationic hydrophilic polymers and the zwitterionic lipid bilayer of mammalian cells, novel antibacterial polymers have been shown to exhibit higher biocompatibility than conventional cationic hydrophobic polymers. Nevertheless, few eco-friendly synthetic strategies for producing antibacterial polymers with cationic and hydrophilic characteristics have been developed. Moreover, it is still challenging to obtain cationic polymers that exhibit bactericidal activity and are biocompatible with mammalian cells.

Cellulose is a naturally abundant polysaccharide that exists in nature as crystalline fibers.^17,18^ Recently, nanocelluloses, including cellulose nanofibers and cellulose nanocrystals, which can be produced by chemical and/or mechanical treatments of cellulose-containing natural sources, have received increasing attention as sustainable nanomaterials with desirable stability, mechanical stiffness, biodegradability, and biocompatibility.^17−20^ These characteristic properties of nanocelluloses suggest the potential for use in biomedical applications. In terms of antibacterial materials, surface-aminated cellulose nanofibers and nanocrystals have been investigated and are similar to antibacterial β-glucans such as chitosan composed of d-glucosamine repeating units.^21^ However, the reactivity of hydroxyl groups on the solid surface of nanocelluloses is generally poor, and the regiospecific modification of hydroxyl groups for precise control of the surface structures and functions is complicated. Therefore, the design and synthesis of surface-functionalized nanocelluloses are frequently complicated and laborious.

As alternatives to naturally derived nanocelluloses, crystalline cellulose assemblies can be artificially built from chemically synthesized cellulose molecules, thereby producing synthetic nanocelluloses in a bottom-up manner.^22−26^ Although the chemical synthesis of cellulose molecules through organic or polymer synthesis is generally laborious due to the need to protect/deprotect hydroxyl groups, enzyme-catalyzed polymerization can synthesize cellulose molecules simply using nonprotected saccharides in a single reaction solution. Enzyme-catalyzed polymerization is considered eco-friendly because it proceeds in aqueous solvents under mild conditions (namely, biological temperature, neutral pH, and ambient pressure). Furthermore, the synthesized cellulose molecules self-assemble in situ into synthetic nanocelluloses in the reaction solution through the insolubilization of the products in aqueous solvents. Because cellulose molecules with a degree of polymerization (DP) value greater than 6 are hardly dissolved in water under ambient conditions,^27−29^ the cellulose molecules obtained by enzyme-catalyzed polymerization are normally oligomers.

Remarkably, one-terminally functionalized cellulose molecules can be synthesized through the cellodextrin phosphorylase (CDP)-catalyzed oligomerization of α-d-glucose 1-phosphate (αG1P) monomers (that is, glucosyl donors) from a d-glucose primer (that is, a glucosyl acceptor at the initiation reaction step) with a functional group at the reducing end.^24^ In fact, the synthesis of cellulose molecules one-terminally functionalized with amino,^30^ azido,^31,32^ vinyl,^33^ thiol,^34,35^ phenolic,^36^ oligo(ethylene glycol),^37,38^ or alkyl^39^ groups and the production of their crystalline assemblies have been reported. We previously reported the synthesis of one-terminally aminated cellulose and its self-assembly into sheet-shaped nanoparticles.^30,40^ The one-terminally aminated cellulose chains aligned perpendicularly to the base plane of the nanosheets in an antiparallel molecular arrangement (namely, cellulose II allomorph), displaying the amino groups on the assembly surfaces. These cationic polymer assemblies exhibited high cytocompatibility, probably due to the hydrophilicity of the cellulose chains.^30,40^

**Figure 1:**
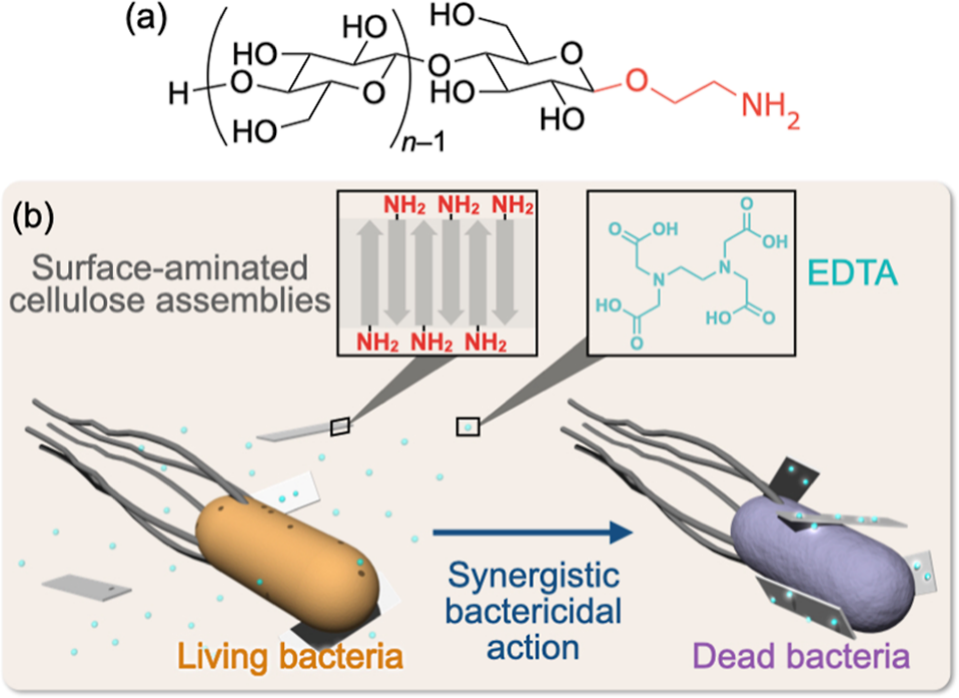
Schematic illustration of this study. (a) Chemical structure of one-terminally aminated cellulose oligomers. (b) Antibacterial action of the surface-aminated cellulose assemblies with EDTA.

We hypothesized that the surface-aminated cellulose assemblies could be eco-friendly antibacterial polymeric nanomaterials with biocompatibility with mammalian cells. In this study, we investigated the activities of surface-aminated cellulose assemblies with a nanosheet morphology against bacteria (Figure 1). Plain cellulose assemblies without amino groups on the surface were used as a reference. *Escherichia coli* (*E. coli*) and *Staphylococcus aureus* (*S. aureus*) were used as representative Gram-negative and -positive bacteria, respectively. Antibacterial properties were evaluated by the growth curve and colony counting assays. As it is well established that divalent cations (such as Ca^2+^ or Mg^2+^) significantly increase the survival rate of bacteria against cationic peptides,^41^ EDTA was used concurrently with the cellulose assemblies to evaluate their synergistic effects. Complex formation between the assemblies and bacteria was investigated microscopically. The interaction of the assembly with an LPS Ra monolayer as a model of a Gram-negative bacteria surface in the presence or absence of EDTA was analyzed by a quartz crystal microbalance with dissipation monitoring (QCM-D) technique. This study will pave a new way to develop synthetic nanocellulose-based antibacterial materials with eco-friendliness, biodegradability, and biocompatibility.

## 2 Experimental Section

### 2.1 Materials

Crystalline assemblies composed of one-terminally aminated and plain cellulose oligomers were prepared by enzymatic reactions using CDP derived from *Acetivibrio thermocellus* DSM 1313 according to our previous reports.^30,42^ CDP was prepared by introducing the gene sequence into the restriction enzyme site NcoI-XhoI of pET-28a(+) with a His-tag on the C-terminal side [host cell: *E. coli* BL21-Gold (DE3)]. For the synthesis of one-terminally aminated cellulose oligomers, 200 mM αG1P monomers and 50 mM 2-aminoethyl-β-d-glucoside primers were incubated with CDP in a 500 mM 4-(2-hydroxyethyl)-1-piperazineethanesulfonic acid buffer solution (pH 7.5) containing 50 μM EDTA for 3 d at 60 °C. The CDP concentration was adjusted to that for synthesizing plain cellulose oligomers with a monomer conversion of approximately 35% in the absence of EDTA.^42^ Notably, the use of EDTA in this study increased monomer conversion from 20 to 45% for the synthesis of one-terminally aminated cellulose oligomers. *n*-Octadecyltrimethoxysilane (ODTMS) was purchased from Fluorochem Ltd. (Derbyshire, UK). LPS Ra was a generous gift from Prof. K. Brandenburg and Prof. Gutsmann (Research Center Borstel, Germany). LPS Ra was extracted from *Salmonella enterica* (serovar Minnesota) strain R60, and the purified samples were lyophilized according to the protocol described previously.^43^ Unless otherwise stated, reagents were purchased from Nacalai Tesque, Inc. (Kyoto, Japan). Ultrapure water (more than 18.2 MΩ cm) supplied by a Milli-Q Advantage A-10 (Merck Millipore) was used throughout the study.

### 2.2 Structural Characterization

For ^1^H nuclear magnetic resonance (NMR) spectrometry, cellulose samples were dissolved in 4% sodium deuteroxide/deuterium oxide. An AVANCE III HD spectrometer (500 MHz, Bruker Corp., Massachusetts, USA) recorded the spectra at room temperature. The residual signals of water (δ = 4.79) as an internal standard were used to calibrate the spectra. For matrix-assisted laser desorption/ionization time-of-flight (MALDI-TOF) mass spectrometry, cellulose samples were dispersed in acetonitrile/water (1/1, v/v) containing 2,5-dihydroxybenzoic acid (2 mg mL^–1^) and trifluoroacetic acid [0.01% (v/v)]. The dispersions were deposited onto a sample target plate and dried under ambient conditions. An AXIMA-performance mass spectrometer (Shimadzu Corporation, Kyoto, Japan) equipped with a nitrogen laser (λ = 337 nm) and capable of pulsed ion extraction using linear/positive mode measured the spectra at room temperature, followed by calibration with bradykinin (757.3997 Da), P_14_R (1533.8582 Da), and ACTH (2465.1989 Da). For attenuated total reflection-Fourier transform infrared (ATR–FTIR) absorption spectrometry, cellulose samples with and without EDTA in a powder state were deposited on the prism surface of an ATR attachment (ATR PRO450-S, Jasco Corp., Tokyo, Japan). The cellulose samples with EDTA were prepared as follows: 0.1% (w/v) surface-aminated cellulose assemblies were mixed with 500 μM EDTA, incubated for 48 h at 37 °C, purified with water through three centrifugation/redispersion cycles, and freeze-dried. An FT/IR-4100 FTIR spectrometer (Jasco Corporation, Tokyo, Japan) recorded the spectra at a cumulative number of 100 and a resolution of 2.0 cm^–1^ under ambient conditions. For atomic force microscopy (AFM), aqueous dispersions of cellulose samples were spin-cast on mica and then observed using an SPM-9700HT instrument (Shimadzu Corp., Kyoto, Japan) in the tapping mode.

### 2.3 Antibacterial Assays

Cellulose samples were sterilized by the autoclave treatment (20 min at 120 °C) before antibacterial assays. Unless otherwise stated, *E. coli* ER2738 or *S. aureus* Rosenbach, 1884 [4.3 × 10^7^ colony forming unit (cfu) mL^–1^] was mixed with surface-aminated or plain cellulose assemblies in the absence or presence of EDTA (100 μM) in 200 μL of a Dulbecco’s phosphate-buffered saline (PBS) solution (137 mM sodium chloride, 8.1 mM disodium hydrogen phosphate, 2.7 mM potassium chloride, 1.5 mM potassium dihydrogen phosphate) in a polystyrene-made 96-well plate with flat bottom (MS-3096F, Sumitomo Bakelite Co., Ltd., Tokyo, Japan). The mixed solutions were incubated with shaking (250 rpm) in a Shaking Incubator MyBLP2S (AS ONE Corporation, Osaka, Japan) for 48 h at 37 °C. To obtain the growth curves of the bacteria, the incubated solutions were diluted 100 times with LB medium prepared from LB Broth (Lennox) (ForMedium, Norfolk, UK). Then, the optical density at 600 nm for 100 μL of the diluted solutions was measured every 10 min with shaking (543 rpm) using a BioTek Synergy H1 microplate reader (Agilent Technologies, Inc., California, USA) during incubation for 24 h at 37 °C. Then, the bacterial suspensions were diluted appropriately and applied to LB-AGAR plates prepared from LB-AGAR (Lennox) (ForMedium, Norfolk, UK) for 24 h at 37 °C for colony counting assays.

### 2.4 Fluorescence Microscopy

The mixed solutions containing *E. coli* ER2738 (4.3 × 10^7^ cfu mL^–1^), surface-aminated or plain cellulose assemblies [0.02% (w/v)], and EDTA (100 μM) were diluted 10-fold with PBS either immediately after mixing or after incubation for 48 h at 37 °C. Eight microliters of the diluted solutions were placed on a glass slide, mixed with 1 μL of Calcofluor White Stain (Merck KGaA) and 10% KOH aqueous solution, and then covered with a cover glass. After incubation for 1 min under ambient conditions, fluorescence microscopy images were obtained using a fluorescence microscope (Eclipse LV100ND, Nikon, Tokyo, Japan) with excitation, dichroic mirror, and barrier filter wavelengths of 330–380, 400, and 420 nm, respectively, at room temperature.

### 2.5 QCM-D Analysis

The interactions of the surface-aminated cellulose assemblies with the outer surface of Gram-negative bacteria in the absence and presence of EDTA (100 μM) were monitored by QCM-D. The changes in Δ*f* and Δ*D* were recorded at 37 °C using a Q-Sense E4 instrument (Gothenburg, Sweden). QCM-D crystals coated with silicon dioxide (SiO_2_) were cleaned in 10 mM sodium dodecyl sulfate, rinsed in water, and kept in a UV–ozone chamber for 20 min before each measurement. All measurements were recorded at every odd overtone up to the 13th overtone throughout the study. For the analysis, we used the data from the 5th, 7th, 9th, and 11th overtones. In this paper, the normalized value at the fifth overtone is presented for Δ*f*. An LPS Ra monolayer was deposited on the substrate precoated with a hydrophobic ODTMS monolayer.^44,45^ Following the establishment of a baseline in PBS buffer containing no EDTA (EDTA-free buffer), LPS Ra suspended in EDTA-free buffer (0.1 mg mL^–1^) was injected into the cell at a flow rate of 20 μL min^–1^ for 2 h. Once Δ*f* and Δ*D* became stable, unbound LPS molecules were washed off by EDTA-free or EDTA-loaded buffer. After confirming that the signal showed no drift for >10 min, the saturation level was recorded for each condition. The surface-aminated cellulose assemblies suspended in EDTA-free or EDTA-loaded buffer were injected after confirming that the system reached a steady state, and the changes in the resonant frequency shift (Δ*f*) and energy dissipation shift (Δ*D*) were monitored over time. As the change in the dissipation caused by the deposition of the LPS Ra monolayer was not negligible, Δ*D* > 1 × 10^–6^ (see the Results Section), the obtained data were fitted with the Voigt model.^46,47^ The solvent density at 37 °C (1001 kg m^–3^) and the LPS layer density (1000 kg m^–3^) were held constant during the fitting.

### 2.6 Hemolysis Assay

One percent blood erythrocytes (sheep, unlabeled, Cosmo Bio Co., Ltd., Tokyo, Japan) were incubated with adequate concentrations of surface-aminated or plain cellulose assemblies in 150 μL of PBS for 4 h at 37 °C with shaking at 567 rpm using a shaking incubator (MyBLP2S, AS ONE CORPORATION, Osaka, Japan) in a polystyrene-made 96-well plate with a flat bottom (MS-3096F, Sumitomo Bakelite Co., Ltd., Tokyo, Japan). For a positive control, 10% Triton X-100 was used instead of the cellulose assemblies. For a negative control, blood erythrocytes were incubated only in PBS. After incubation, the solutions were centrifuged at 20,400*g* for 10 min at 25 °C. Then, the absorbance at 400 nm (*A*_400_) for 100 μL of the supernatants in 96-well plates was measured using a BioTek Synergy H1 microplate reader at room temperature. Hemolysis was calculated from the following equation.
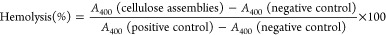


## 3 Results and Discussion

### 3.1 Preparation of Cellulose Assemblies

The surface-aminated and plain cellulose assemblies were prepared by CDP-catalyzed oligomerizations of αG1P monomers from the appropriate primers. Structural characterization confirmed the production of crystalline assemblies composed of one-terminally aminated and plain cellulose oligomers (see Figures S1 and S2), as we previously reported.^30,48^ The average DP values of the cellulose moieties for the one-terminally aminated and plain oligomers were estimated to be approximately 9–10 and 10 by ^1^H NMR spectrometry, respectively.

### 3.2 Bacteriostatic Activity against Gram-Negative *E. coli*

**Figure 2:**
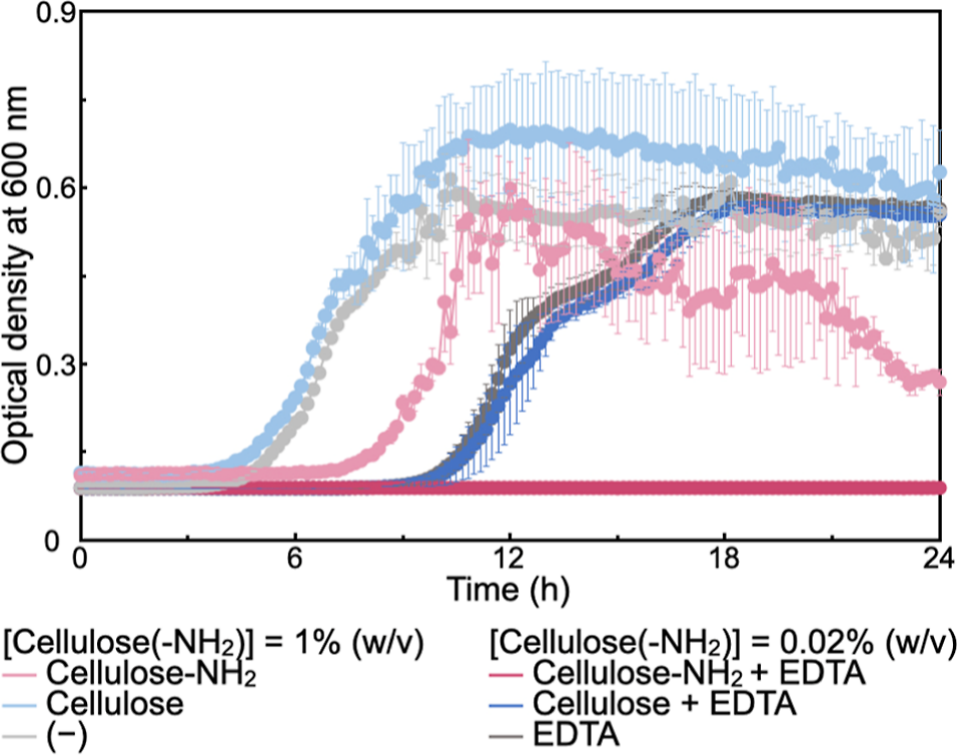
Time course of the optical densities of *E. coli* suspensions during incubation with and without surface-aminated and plain cellulose assemblies in the absence or presence of EDTA. The experiments were repeated three times, and the error bars correspond to the standard deviation.

To analyze the bacteriostatic activities of the surface-aminated and plain cellulose assemblies, Gram-negative *E. coli* (4.3 × 10^7^ cfu mL^–1^) was incubated with these assemblies [0.02% (w/v)] in the absence or presence of EDTA (100 μM) for 48 h at 37 °C, and then, the growth curves of *E. coli* were obtained (Figure 2). An increase in optical density (namely, the turbidity of the solutions) for growth curves indicates that the concentration of bacteria exceeds a certain level. In the absence of any additives (namely, cellulose assemblies and EDTA), the optical density gradually increased after incubation for approximately 5 h, indicating the growth of *E. coli*. In the presence of the plain cellulose assemblies without EDTA, the growth curve was almost identical with that without additives. This observation suggests that the plain cellulose assemblies have no impact on *E. coli* under these conditions, indicating that the plain cellulose assemblies have no or negligible antibacterial properties. Significantly, in the presence of the surface-aminated cellulose assemblies without EDTA, the optical density gradually increased after the incubation for approximately 7 h. Therefore, the time at which optical density started to increase was slightly delayed (approximately 2 h delay) compared with the times obtained under the previous two conditions, indicating that the surface-aminated cellulose assembly had a certain level of antibacterial properties.

Moreover, EDTA delayed the growth of *E. coli* for approximately 4 h (Figure 2), indicating a certain antibacterial effect of EDTA under these conditions. In the presence of the plain cellulose assemblies and EDTA, the time to initiate the increase in the optical density was almost the same as that in the presence of EDTA alone, further suggesting that the plain cellulose assemblies had no detectable antibacterial properties. Remarkably, in the presence of the surface-aminated cellulose assemblies and EDTA, the optical density did not increase during incubation for 24 h, showing suppression of the growth of *E. coli*. This observation indicates a synergistic effect between the surface-aminated cellulose assemblies and EDTA for the bacteriostatic activities.

### 3.3 Bactericidal Activity against Gram-Negative *E. coli*

**Figure 3:**
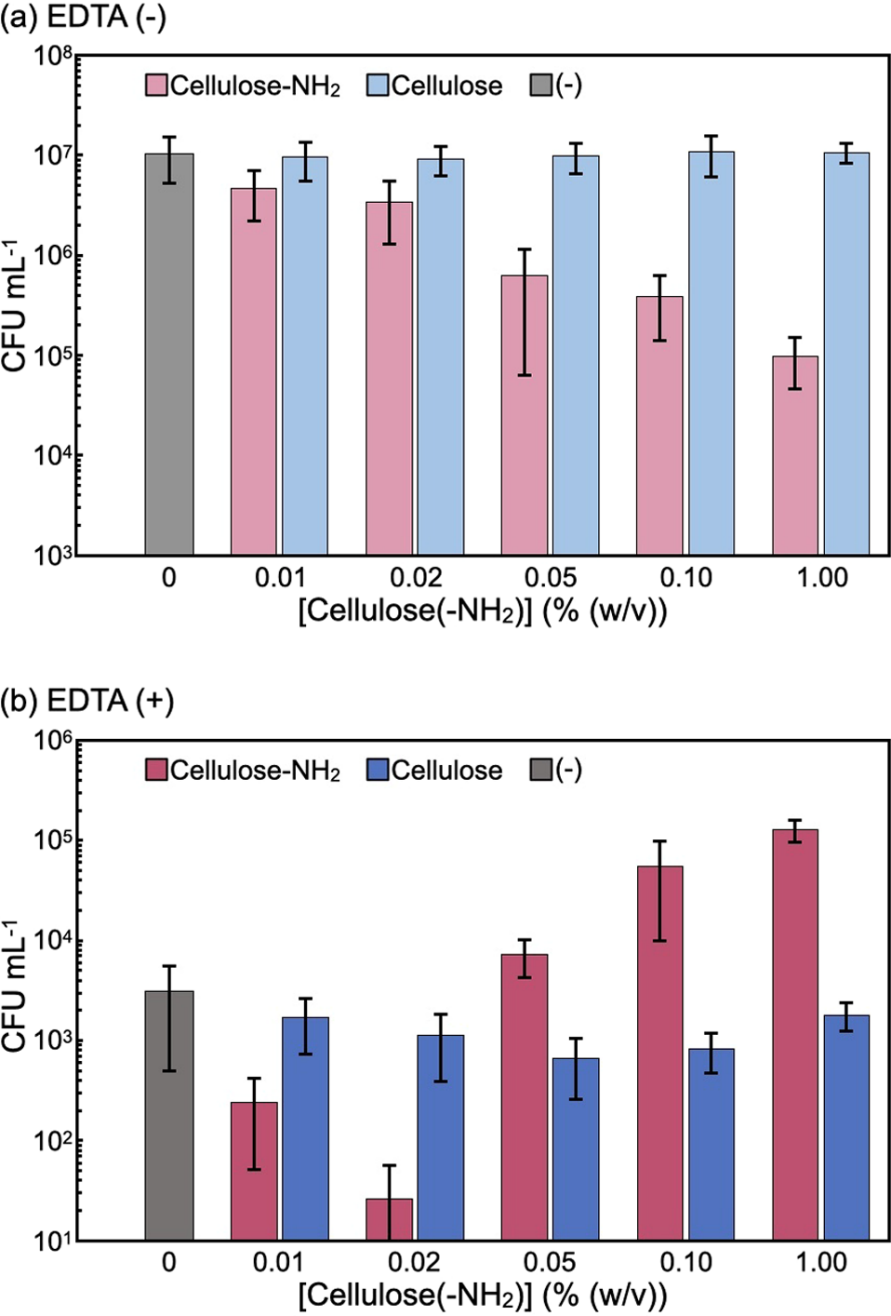
Colony counting assays for *E. coli* suspensions after incubation with the surface-aminated or plain cellulose assemblies in the (a) absence and (b) presence of EDTA at different assembly concentrations for 48 h. The experiments were repeated three times, and the error bars correspond to the standard deviation.

To analyze bactericidal activities, *E. coli* (4.3 × 10^7^ cfu mL^–1^) was subjected to a colony counting assay after incubation with the surface-aminated or plain cellulose assemblies in the absence or presence of EDTA (100 μM) for 48 h at 37 °C. To systematically evaluate the synergistic effects between the cellulose assemblies and EDTA, the concentration of the cellulose assemblies was varied from 0 to 1% (w/v) at a constant concentration of EDTA (Figure 3). In the presence of the plain cellulose assemblies without EDTA, cfu values were almost the same as those of the control without the assemblies, irrespective of the concentration of the assemblies, confirming that the plain cellulose assemblies do not have any antibacterial properties under the conditions tested (Figure 3a). On the other hand, in the presence of surface-aminated cellulose assemblies without EDTA, viable bacteria decreased with increasing assembly concentration. In fact, the cfu value at an assembly concentration of 1% (w/v) corresponded to 1% of that obtained for the control without the assemblies. These observations were consistent with the results obtained from the aforementioned growth curve analysis. The surface-aminated cellulose assemblies were likely to exhibit antibacterial activity via destabilization of the cell wall of *E. coli* through electrostatic interactions between the cationic assemblies and anionic LPS molecules existing in the outer membrane.^49^ Even after decreasing the incubation time from 48 to 24 h, a similar trend was observed (Figure S3a). Nevertheless, the degree of reduction in viable bacteria by the surface-aminated cellulose assemblies decreased; for instance, the cfu value at an assembly concentration of 1% (w/v) was 15% of that for the control without the assemblies. Incubation of *E. coli* in PBS without any additives for 24 and 48 h slightly decreased cfu values to 33 and 17% of that before incubation, respectively (Figure S4). In summary, the cationic assemblies were shown to kill bacterial cells gradually during 48 h of incubation.

In the presence of EDTA without cellulose assemblies, the cfu value was 0.03% of that without cellulose assemblies and EDTA (Figure 3b), indicating the antibacterial properties of EDTA, which destabilized the cell walls of *E. coli* by removing metal ions from LPS molecules.^50−52^ In the presence of EDTA and different concentrations of the plain cellulose assemblies, the cfu values hardly changed, confirming that the nonionic assemblies did not have antibacterial properties under these conditions. In contrast, in the presence of surface-aminated cellulose assemblies and EDTA, synergistic effects were observed. For example, at an assembly concentration of 0.02% (w/v), cfu values with and without EDTA corresponded to 0.0002 and 14% of those without cellulose assemblies and EDTA, respectively. A similar trend was observed even after incubation for 24 h (Figure S3). Considering the decrease in cfu caused by EDTA alone (0.03%), synergy between the cationic assemblies and EDTA for bactericidal actions clearly occurred.

For the synergistic effects, the ratio of the surface-aminated cellulose assemblies and EDTA was found to be important. At an EDTA concentration of 100 μM, increasing the concentration of the cationic assemblies from 0 to 0.01 or 0.02% (w/v) decreased the cfu values (Figure 3b). However, further increases in cationic assembly concentration significantly increased cfu values. In fact, the cfu value at 0.05% (w/v) cationic assemblies was comparable to that with EDTA alone, and the values at 0.1% (w/v) and 1% (w/v) were even higher, indicating that the antibacterial actions of EDTA were suppressed by sufficient concentrations of the surface-aminated cellulose assemblies. This may result from a decrease in the effective concentration of EDTA due to the electrostatic adsorption of EDTA onto the surface-aminated cellulose assemblies. In fact, the potential for complex formation between the surface-aminated cellulose assemblies and EDTA was confirmed by ATR–FTIR absorption spectrometry (Figure S5). To interpret the dependence of the antibacterial actions on the assembly concentration, the number ratios between the total carboxyl groups of EDTA and the total amino groups of the surface-aminated cellulose assemblies (that is, COOH/NH_2_) at different assembly concentrations were estimated and are shown in Table S1. The estimation suggests an appropriate COOH/NH_2_ value for synergistic effects. Approximately three times more carboxyl groups than amino groups, where the surface-aminated cellulose assemblies were 0.02% (w/v), appeared to be nearly optimal at the 100 μM EDTA. In other words, because EDTA has four carboxyl groups, the number of amino groups was comparable to or slightly greater than the number of EDTA molecules under nearly optimal conditions.

**Figure 4:**
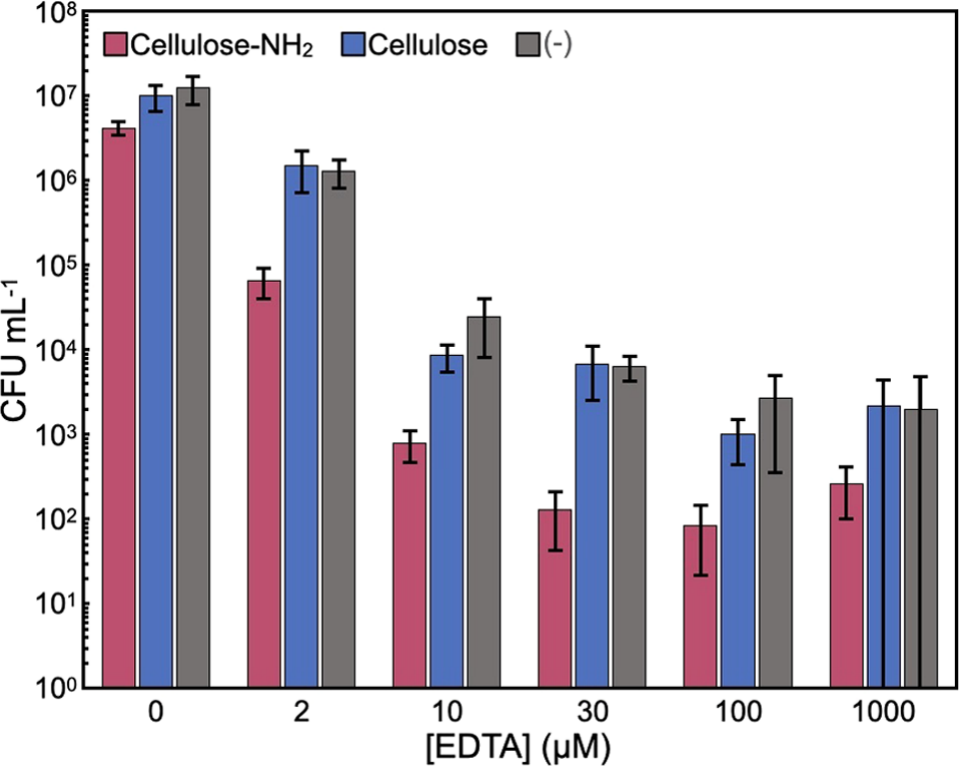
Colony counting assays of *E. coli* suspensions after incubation with surface-aminated or plain cellulose assemblies at different EDTA concentrations. The experiments were repeated three times, and the error bars correspond to the standard deviation.

To gain further insights into the synergistic effects, the EDTA concentration was changed from 0 to 1000 μM at a constant concentration [0.02% (w/v)] of cellulose assemblies (Figure 4). At all EDTA concentrations, cfu values were hardly affected by the presence of the plain cellulose assemblies, further confirming that the nonionic assemblies did not have antibacterial properties under these conditions. On the other hand, cfu values were minimal at 30 and 100 μM EDTA, where the COOH/NH_2_ values were 0.96 and 3.2, respectively. Although the optimal COOH/NH_2_ values might be slightly different between the dependences of the surface-aminated cellulose assembly and EDTA concentrations, it was proposed that there were appropriate COOH/NH_2_ values for the synergistic effects. Consequently, it was found that the concurrent use of the surface-aminated cellulose assemblies and EDTA led to bactericidal actions.

### 3.4 Complex Formation between Cationic Cellulose Assemblies and *E. coli*

Interactions of cellulose assemblies with *E. coli* (that is, complex formation) in the absence or presence of EDTA were microscopically observed to understand the underlying mechanism of the synergistic effect between the surface-aminated cellulose assemblies and EDTA. The concentrations of cellulose assemblies, EDTA, and *E. coli* were set to 0.02% (w/v), 100 μM, and 4.3 × 10^7^ cfu mL^–1^, respectively, and the mixed solutions were incubated for 48 h at 37 °C. These conditions were nearly optimal for the synergistic effect. Then, the cellulose assemblies were stained with Calcofluor White M2R for fluorescence microscopy. For the solutions containing only the surface-aminated or plain cellulose assemblies, fluorescent objects with some aggregates were observed sparsely (Figure S6a,b), suggesting that most of the cellulose assemblies were well dispersed in the field of view. Unexpectedly, rod-like objects were observed in the solution of *E. coli* alone (Figure S6c), suggesting that *E. coli* was also stained by Calcofluor White M2R. Therefore, the cellulose assemblies were distinguished from *E. coli* based on the difference in their morphologies.

**Figure 5:**
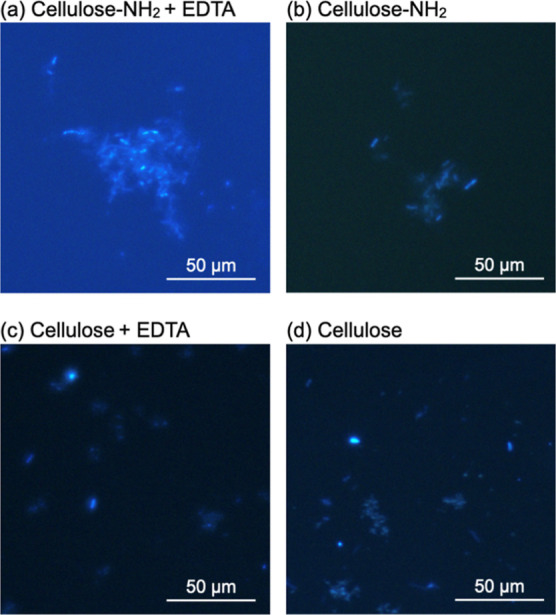
Fluorescence microcopy images of the mixed solutions of (a,b) the surface-aminated or (c,d) plain cellulose assemblies and *E. coli* in the presence (a,c) or absence (b,d) of EDTA after incubation for 48 h.

Figure 5 shows fluorescence microscopy images of the mixture solutions of the surface-aminated or plain cellulose assemblies and *E. coli* in the absence or presence of EDTA. Although the surface-aminated cellulose assemblies and *E. coli* immediately after mixing were observed in dispersed states, after incubation for 48 h, large aggregates were clearly observed. Such large aggregates were absent from the mixed solutions containing the plain cellulose assemblies and *E. coli* even after incubation for 48 h. Judging from the morphologies, the aggregates appeared to be composed of surface-aminated cellulose assemblies and *E. coli*. Because Gram-negative *E. coli* has anionic LPS molecules in the outer membrane,^49^ the cationic assemblies appeared to aggregate with *E. coli* through electrostatic interactions. Furthermore, the images of the mixed solutions containing the surface-aminated cellulose assemblies and *E. coli* were similar in the absence and presence of EDTA, suggesting that EDTA hardly affects aggregate formation under these conditions. This observation suggests that aggregate formation was not solely responsible for the bactericidal actions of the surface-aminated cellulose assemblies and EDTA. Consequently, the amino groups of the assemblies appeared to be used for electrostatic interactions not only with EDTA (Figure S5a) but also with LPS molecules in the outer membrane of *E. coli*.

Assembling all of the observations together, the synergistic effects between the surface-aminated cellulose assemblies and EDTA for the bactericidal actions are proposed as follows: EDTA molecules remove divalent metal ions from the outer membrane of *E. coli* to reduce its barrier functions. Subsequently, the cationic assemblies destabilize the outer membrane of *E. coli* through electrostatic interactions with anionic LPS molecules in the outer membrane, resulting in bactericidal activity. In this scenario, the enrichment of EDTA molecules on the surfaces of the cationic assemblies via electrostatic adsorption may promote the cooperation between EDTA and the cationic assemblies. Such synergistic effects appeared to cause bactericidal actions, as schematically illustrated in Figure 1.

### 3.5 Interactions between Surface-Aminated Cellulose Assemblies and an LPS Monolayer

To speculate about the interaction between the surface-aminated cellulose assemblies and the cell walls of *E. coli*, the interaction between the cationic assemblies and an artificially prepared LPS Ra monolayer in the absence or presence of EDTA was analyzed by a QCM-D technique equipped with a solution-flow system. An LPS Ra monolayer was prepared on the QCM-D substrate as follows. The injection of LPS Ra suspended in an EDTA-free buffer resulted in a decrease in Δ*f* and an increase in Δ*D* of Δ*f*_LPS_ ≈ −36.8 Hz and Δ*D*_LPS_ ≈ 4.7 × 10^–6^, respectively. The fitting of the data with the Voigt model estimated a layer thickness of *d* ≈ 10.3 nm, an elastic modulus of 75.1 kPa, and a shear viscosity of 1.9 mPa s. The exchange of buffer to EDTA-loaded (100 μM) buffer led to very minor changes in Δ*f*_EDTA_ (1.6 Hz) and Δ*D*_EDTA_ (−0.1 × 10^–6^), respectively. Both Δ*f* and Δ*D* remained stable and showed no change over 2 h, suggesting that the LPS Ra monolayer sustained structural integrity in an EDTA-loaded buffer.

**Figure 6:**
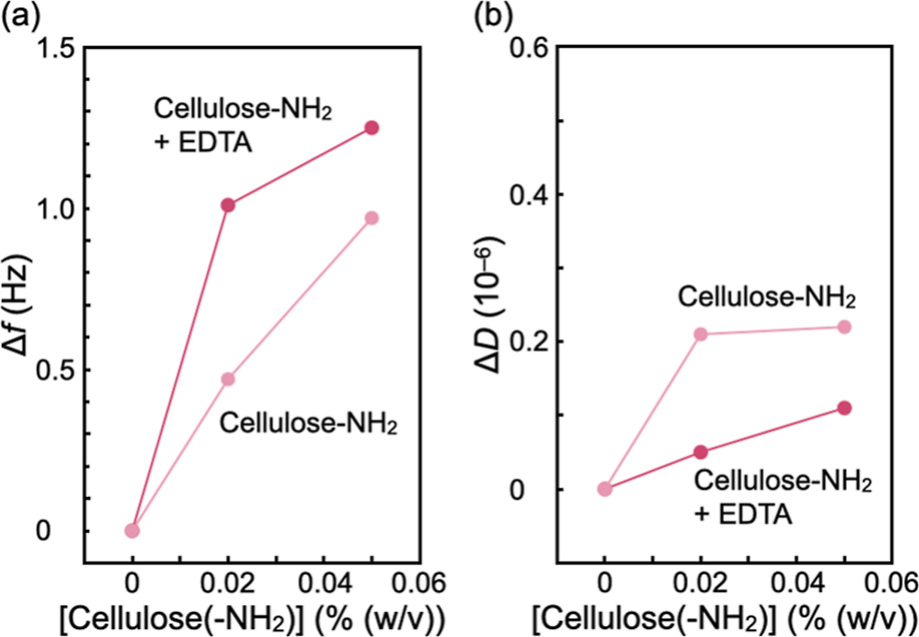
Changes in (a) Δ*f* and (b) Δ*D* with respect to those of the intact monolayers after the LPS Ra monolayer was incubated with the dispersions of the surface-aminated cellulose assemblies for 2 h. Magenta symbols are the data collected in the EDTA-loaded buffer, while pink symbols are in the EDTA-free buffer.

To monitor the initial phase of the interaction between the surface-aminated cellulose assemblies and the LPS Ra monolayer, the changes in Δ*f* and Δ*D* were monitored after 2 h of incubation with suspensions containing 0.02% (w/v) and 0.05% (w/v) cationic assemblies (Figure 6). After each treatment, it took 60–80 min for both Δ*f* and Δ*D* to reach the saturation levels, suggesting that the interaction of cellulose assemblies and the LPS monolayer is driven by the diffusion. As shown in Figure 6a, Δ*f* exhibited a monotonic increase with increasing assembly concentration, suggesting a loss of mass. The change in Δ*f* in EDTA-loaded buffer is more pronounced than that in EDTA-free buffer. The obtained data indicate that the removal of residual divalent cations (Ca^2+^ and Mg^2+^) by EDTA made the LPS Ra monolayer more susceptible to the cationic assemblies, as suggested by the aforementioned antibacterial experimental results (Figures 2–4). Figure 6b shows the changes in Δ*D* after incubation with the cationic assemblies for 2 h. Incubation with the assemblies led to a subtle increase in dissipation, suggesting that the LPS Ra monolayer became slightly more viscous. Although the increase in Δ*D* seems more pronounced in EDTA-free buffer, the changes in dissipation Δ*D* caused by the assemblies were very minor (Δ*D* ≪ 0.5 × 10^–6^), suggesting that the assemblies caused the decrease in mass density but did not significantly change the film viscoelasticity.

After incubation with dispersions of the cationic assemblies [0.05% (w/v)] for 2 h, the samples were rinsed with a blank buffer containing no assemblies to verify whether the changes in Δ*f* caused by the assemblies were reversible. The Δ*f* values slightly increased in both cases but did not recover to the initial levels, suggesting that incubation with the cationic assemblies even for a short time (2 h) caused irreversible damage to the LPS Ra monolayer. The difference in Δ*f* before and after cellulose treatment in the EDTA-loaded buffer (0.9 Hz) was larger than that in the EDTA-free buffer (0.6 Hz), indicating that more LPS Ra molecules were removed by the cationic assemblies in the EDTA-loaded buffer.

To determine how much the assemblies could damage the LPS Ra, the experimental conditions for the effective bactericidal activity, i.e., incubation with 0.02% (w/v) assemblies and 100 μM EDTA for 24 h, were mimicked (Figure S3b). With respect to the intact monolayer, Δ*f* increased by 15.4 Hz and Δ*D* decreased by 4.7 × 10^–6^, suggesting that the viscoelastic LPS Ra monolayer was destroyed by the cationic assemblies, resulting in bactericidal activity. Previously, Herrmann et al. measured the viscoelastic response of the LPS Ra monolayer to a cationic antibacterial peptide (fish protamine) at the air/water interface. The simultaneously measured storage and loss moduli before and after the addition of protamine indicated that the LPS Ra molecules form two-dimensional gels in the presence of Ca^2+^, which protects the LPS layer from adsorption and membrane destruction by protamine. In contrast, the LPS Ra monolayer could not sustain its structural and mechanical integrity in the absence of Ca^2+^.^53^ Moreover, the coarse-grained Monte Carlo simulation suggested that Ca^2+^ ions bound to the negatively charged core saccharides form an electrostatic potential barrier against the adsorption of protamine.^54^ In fact, the higher susceptibility of LPS monolayers to cationic assemblies in the absence of divalent cations and hence in the presence of EDTA seems to agree very well with previous reports on the interactions of LPS monolayers with positively charged antibacterial peptides^53,54^ and antiseptic peptides,^55^ as well as with the utility of commercial sanitizers containing cationic surfactants.^56^

### 3.6 Activity against Gram-Positive *S. aureus*

**Figure 7:**
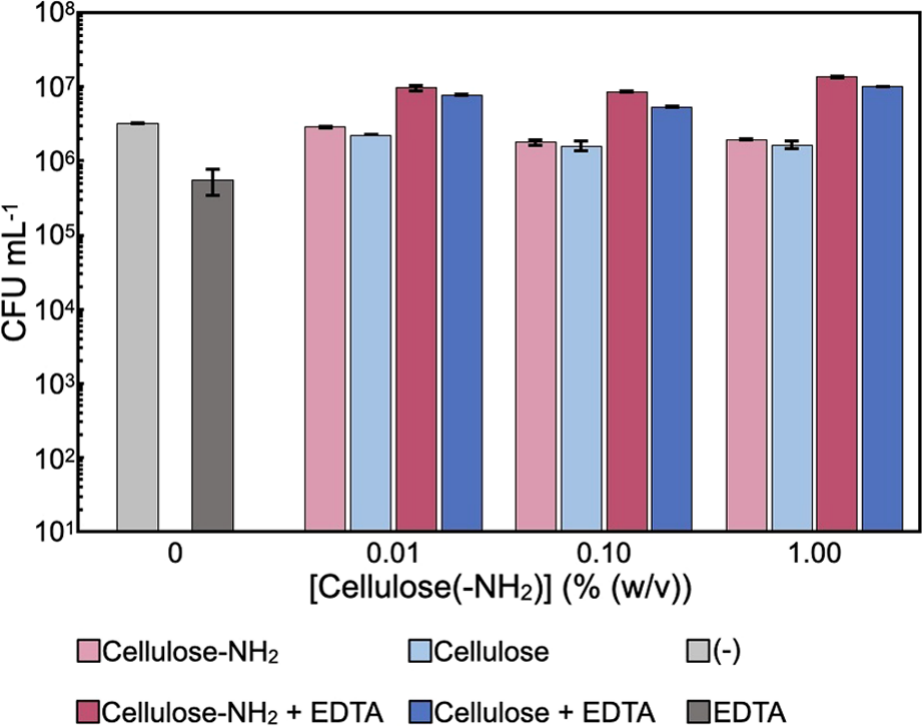
Colony counting assays for *S. aureus* suspensions after incubation with the surface-aminated or plain cellulose assemblies in the presence of EDTA at different assembly concentrations for 48 h. The experiments were repeated three times, and the error bars correspond to the standard deviation.

For comparison with Gram-negative *E. coli*, activities against Gram-positive *S. aureus* at different cellulose concentrations in the presence of EDTA (100 μM) were similarly analyzed by colony counting assays (Figure 7). Importantly, no antibacterial properties of cellulose assemblies against *S. aureus* were observed under any conditions. This result is reasonable when we consider the fact that Gram-positive bacteria do not have an outer membrane containing LPS molecules, which may be essential components for the antibacterial properties of surface-aminated cellulose assemblies and EDTA. Therefore, it seems that the antibacterial properties of the surface-aminated cellulose assembly and its synergy with EDTA are specific for Gram-negative bacteria.

### 3.7 Cytocompatibility Testing by Hemolysis Assays

We previously revealed high cytocompatibility of the surface-aminated cellulose assemblies using a human cancer cell line, namely, HeLa.^30^ On the other hand, hemolysis induced by antibacterial polymers is also an important aspect of cytocompatibility assessment.^57^ Thus, hemolysis assays were performed for the surface-aminated and plain cellulose assemblies in the absence or presence of EDTA (100 μM) to reveal the cytocompatibility of the assemblies (Table 1). Significantly, hemolysis was hardly observed under all conditions, suggesting that the surface-aminated or plain cellulose assemblies and their mixtures with EDTA exhibited no noticeable cytotoxicity. Therefore, the surface-aminated cellulose assemblies and their mixture with EDTA had potential as superior antibacterial agents that do not destroy red blood cells.

**Table 1:** Hemolysis by Surface-Aminated and Plain Cellulose Assemblies in the Absence or Presence of EDTA

cellulose assembly	EDTA	hemolysis (%)		
		concentration of cellulose assembly [% (w/v)]		
		0.01	0.1	1
surface-aminated cellulose assemblies	+	0.2 ± 0.2	0.2 ± 0.5	0.5 ± 0.4
surface-aminated cellulose assemblies	–	–0.6 ± 0.2	0.0 ± 0.7	1.4 ± 1.3
plain cellulose assemblies	–	–0.3 ± 0.8	–0.6 ± 0.6	1.3 ± 1.4

## 4 Conclusions

The antibacterial properties of surface-aminated cellulose assemblies with nanosheet morphologies and their synergy with an antibacterial metal-chelating agent, EDTA, were investigated. The surface-aminated cellulose assemblies, but not the plain cellulose assemblies, had antibacterial properties against Gram-negative *E. coli* without EDTA. The antibacterial activity in the presence of EDTA was pronounced, indicating a clear synergy between the cationic assemblies and EDTA. No antibacterial activity was observed against Gram-positive *S. aureus*, suggesting that destabilization of the outer membrane of Gram-negative bacteria through electrostatic interactions of the cationic assemblies with anionic LPS molecules in the membrane of bacterial cells contributed to the antibacterial properties. The interaction of the surface-aminated cellulose assemblies with artificially produced LPS Ra monolayers was revealed by a QCM-D analysis. The surface-aminated cellulose assemblies exhibited negligible levels of hemolysis, suggesting a high cytocompatibility.

The enzymatic synthesis of one-terminally functionalized cellulose oligomers and the production of structurally regular crystalline assemblies would contribute to the development of cellulose-based antibacterial materials with eco-friendliness, biodegradability, and biocompatibility for a wide variety of applications. Specifically, the combination of surface-aminated cellulose assemblies and chelators will be useful for the disinfection of healthy skin and even infected wounds. Moreover, our findings suggest that the concurrent use of chelators helps antibacterial cationic polymers and nanoparticles to exhibit bactericidal activities while maintaining biocompatibility.

## Supporting Information Available

Characterization of one-terminally aminated cellulose oligomers; characterization of plain cellulose oligomers; colony counting assays for *E. coli* suspensions after incubation with the surface-aminated or plain cellulose assemblies in the absence and presence of EDTA at different assembly concentrations for 24 h; colony counting assays of *E. coli* suspensions before and after incubation in PBS without any additives for 24 and 48 h; ATR–FTIR absorption spectra of surface-aminated cellulose assemblies after incubation with EDTA for 48 h and EDTA alone; chemical structure of EDTA; fluorescence microscopy images of surface-aminated cellulose assemblies, plain cellulose assemblies, and *E. coli* without any additives; and concentration dependence of surface-aminated cellulose assemblies on COOH/NH_2_ in the presence of 100 μM EDTA (PDF)
