# Supplementary material for: Antibacterial Synthetic Nanocelluloses Synergizing with a Metal-Chelating Agent
Source: ACS Appl Bio Mater. 2023 Nov 15;7(1):246–55. doi: 10.1021/acsabm.3c00846 (PMC10792664; doi:10.1021/acsabm.3c00846)
Supplement: Supplementary file 1 — mt3c00846_si_001.pdf [file mt3c00846_si_001.pdf]

Supporting Information

Antibacterial Synthetic Nanocelluloses Synergizing with a Metal-Chelating Agent

Takeshi Serizawa,[,†] Saeko Yamaguchi,[†] Kai Sugiura,[†] Ramona Marten,[‡,§] Akihisa Yamamoto,[§] Yuuki Hata,[†] Toshiki Sawada,[†] Hiroshi Tanaka,[†] and Motomu Tanaka[‡,§]*

[†] Department of Chemical Science and Engineering, School of Materials and Chemical Technology, Tokyo Institute of Technology, 2-12-1 Ookayama, Meguro-ku, Tokyo 152-8550, Japan

[‡] Physical Chemistry of Biosystems, Institute of Physical Chemistry, Heidelberg University, D69120 Heidelberg, Germany

[§] Center for Integrative Medicine and Physics, Institute for Advanced Study, Kyoto University, 606-8501 Kyoto, Japan

KEYWORDS: cellulose oligomer, crystalline assembly, antibacterial cationic polymer, bactericidal activity, ethylenediaminetetraacetic acid, synergistic effect

*E-mail: serizawa@mac.titech.ac.jp

Contents

Page
Figure S1..S3
Figure S2..S4
Figure S3..S5
Figure S4..S6
Figure S5..S6
Figure S6..S7
Table S1..S7

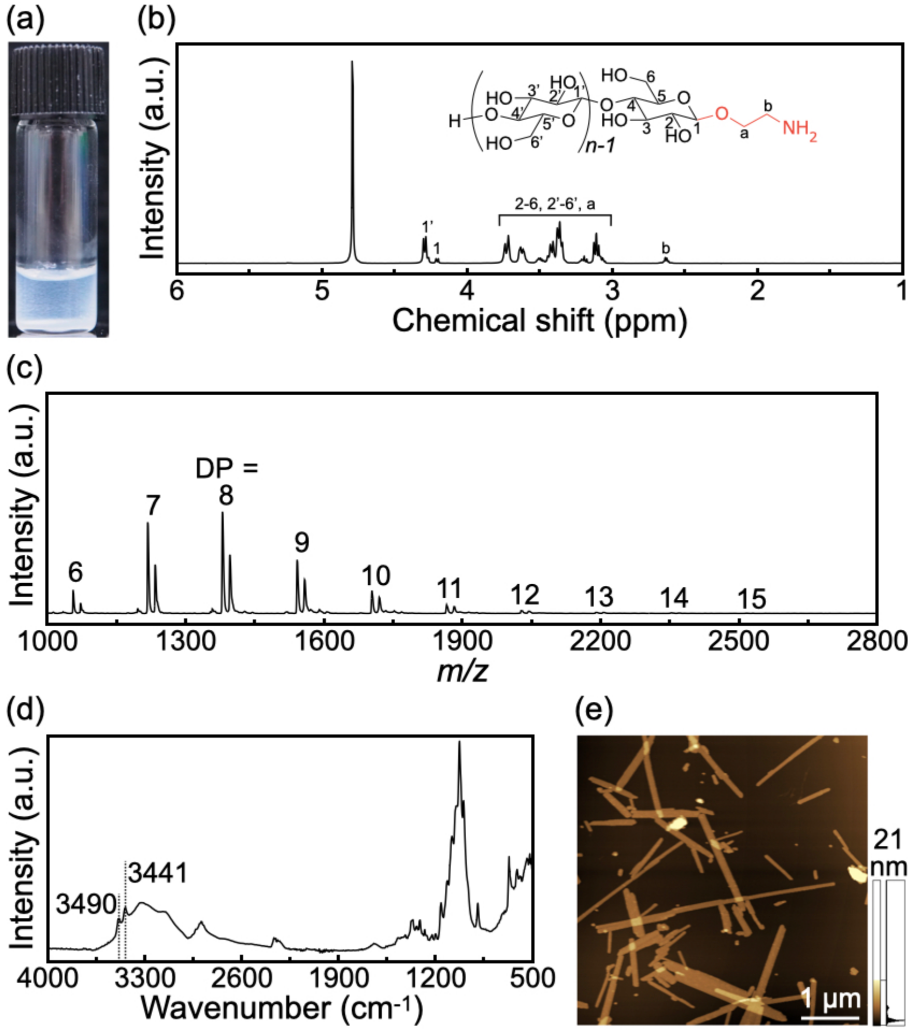

Figure S1. Characterization of one-terminally aminated cellulose oligomers. (a) Photograph of the reaction solution after incubation for 3 days. (b) ^{1}H NMR spectrum, (c) matrix-assisted laser desorption/ionization time-of-flight (MALDI-TOF) mass spectrum, (d) attenuated total reflection-Fourier transform infrared (ATR-FTIR) absorption spectrum, and (e) atomic force microscopy (AFM) image of the oligomers.

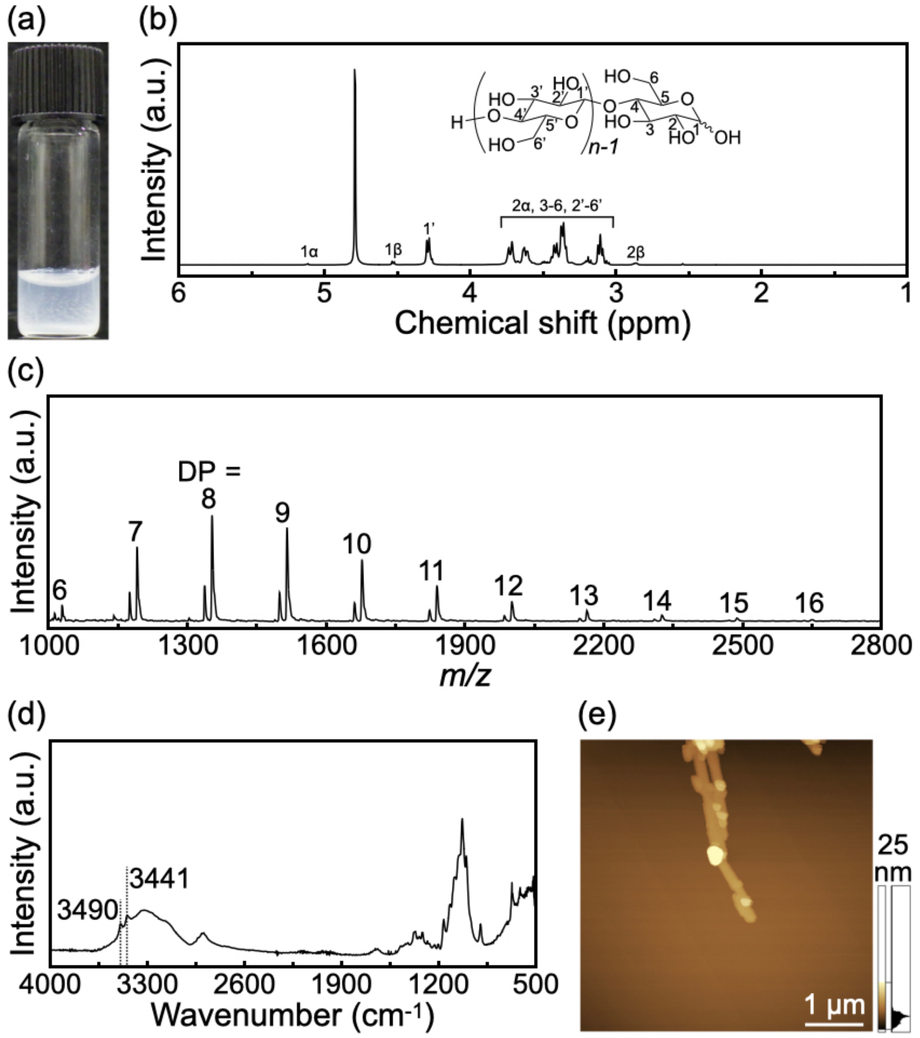

Figure S2. Characterization of plain cellulose oligomers. (a) Photograph of the reaction solution after incubation for 3 days. (b) ^{1}H NMR spectrum, (c) MALDI-TOF mass spectrum, (d) ATR-FTIR absorption spectrum, and (e) AFM image of the oligomers.

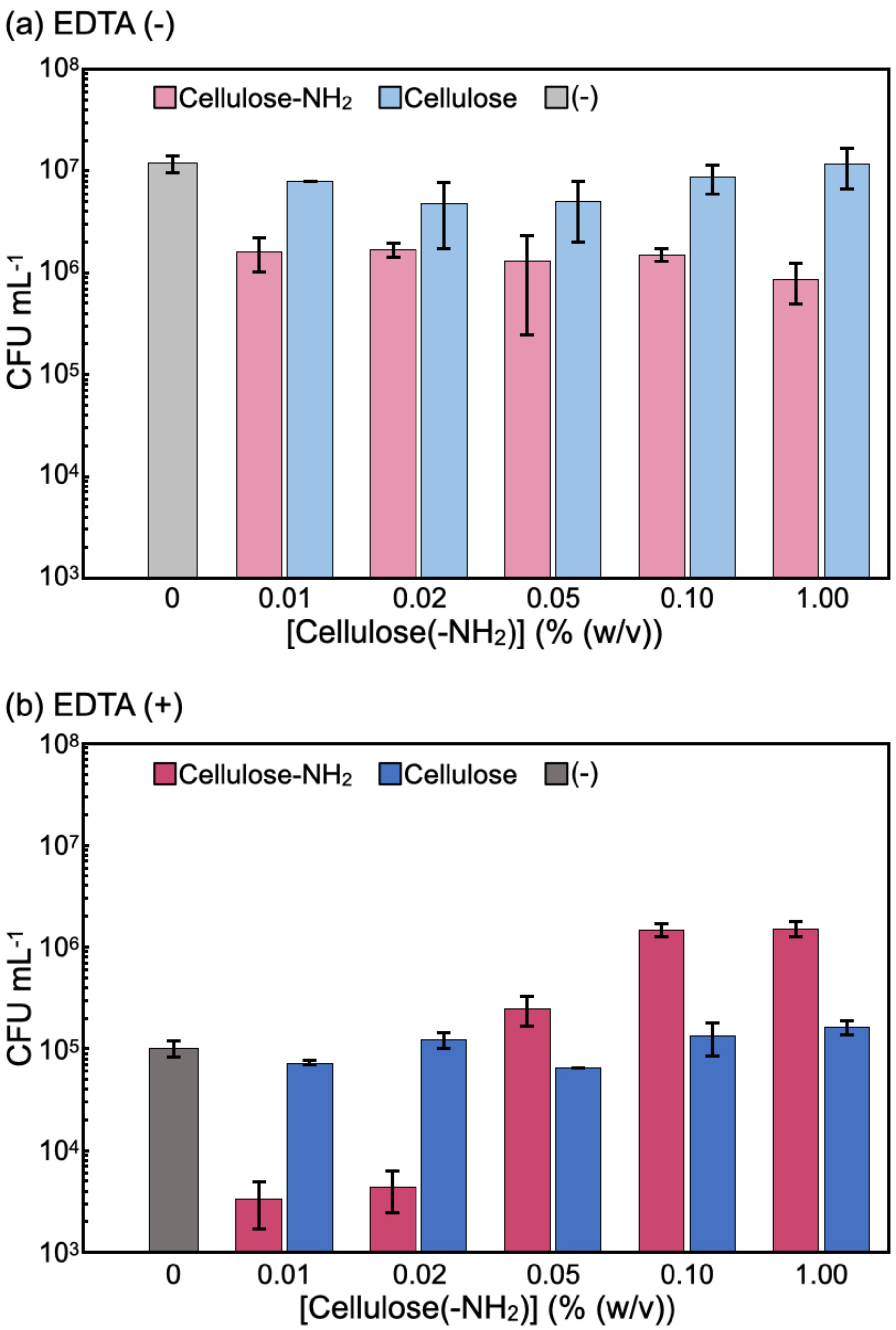

Figure S3. Colony counting assays for *Escherichia coli* (*E. coli*) suspensions after incubation with surface-aminated or plain cellulose assemblies in the (a) absence and (b) presence of ethylenediaminetetraacetic acid (EDTA) at different assembly concentrations for 24 h. The experiments were repeated three times, and the error bars correspond to the standard deviation.

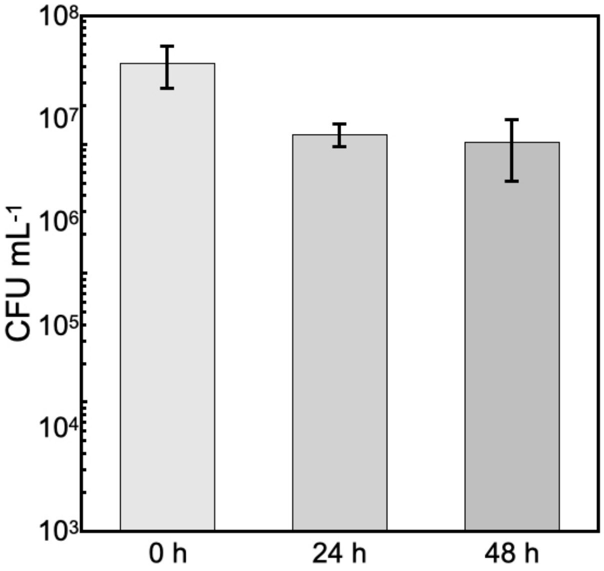

Figure S4. Colony counting assays for *E. coli* suspensions before and after incubation in phosphate-buffered saline without any additives for 24 h and 48 h.

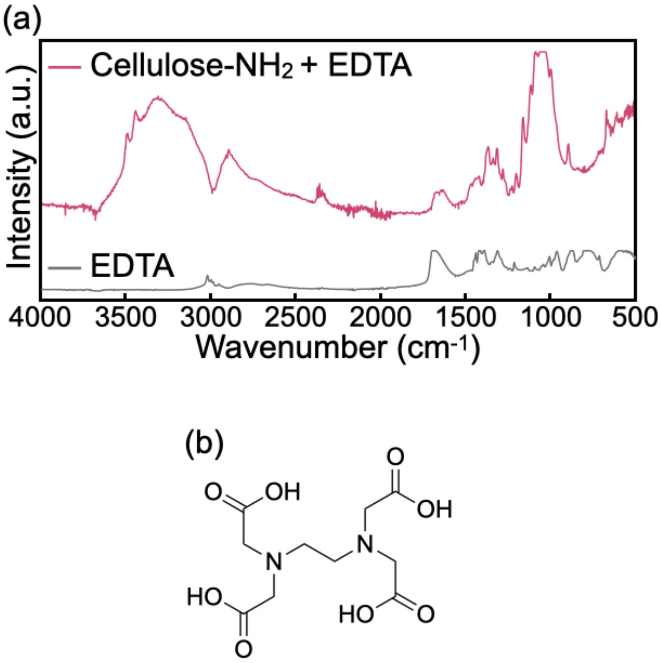

Figure S5. (a) ATR-FTIR absorption spectra of surface-aminated cellulose assemblies after incubation with EDTA for 48 h and EDTA alone. (b) Chemical structure of EDTA.

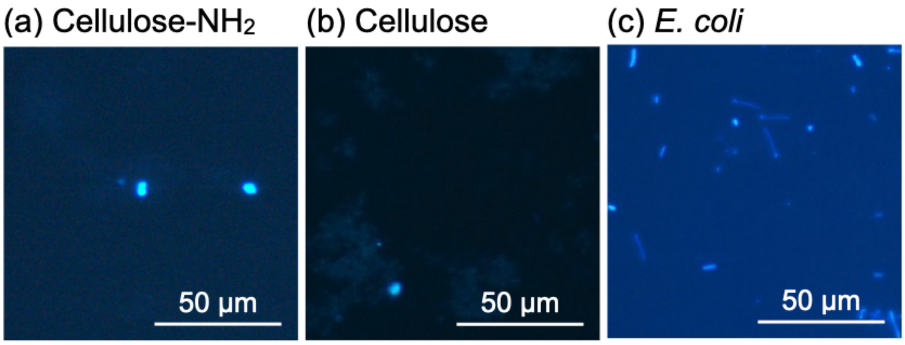

Figure S6. Fluorescence microcopy images of (a) surface-aminated cellulose assemblies, (b) plain cellulose assemblies, and (c) *E. coli* without any additives.

Table S1. Concentration dependence of surface-aminated cellulose assemblies on $COOH/NH_2$ in the presence of 100 μM EDTA.

[Cellulose-NH_2] (% (w/v))	0.01	0.02	0.05	0.10	1.00
Ratio ($COOH/NH_2$)[a]	6.4	3.2	1.3	0.64	0.064

[a]$COOH/NH_2$ values are the number ratios between the total carboxyl groups of EDTA and the total amino groups of the surface-aminated cellulose assemblies.
